# Three New Remedial Preparations

**Published:** 1893-08

**Authors:** W. E. Walker

**Affiliations:** Bay St. Louis, Miss.


AMERICAN JOURNAL

—OF—

DENTAL SCIENCE.

VOL. XXVII. BALTIMORE, AUGUST, 1893. NO. 4.

ARTICLE I.

### THREE NEW REMEDIAL PREPARATIONS.

BY DR. W. E. WALKER, BAY ST. LOUIS, MISS.

Read before the Mississippi State Dental Society, April, 1893.

Among the many new discoveries there are three in materia medica which enables us to employ oxygen and iron in better form than ever before. I have reference to *pyrozone*, *Weld's syrup* of iron chloride, and the bi-palatinoids.

There is probably no one here who has not learned from trying experience the extreme unreliability of peroxide of hydrogen. Notwithstanding all our care in guarding against change of temperature, exposure to light, etc., we too often find our *stock-bottle* of no more value than so much clear water.

McKesson & Robbins, of New York, now offers us, in the three grades of pyrozone, reliable, stable preparations of oxygen in solution of exact strength, which, in the grade indicated by conditions, will be found very valuable in the diagnosis of pus and in the treatment of ulcerations—internal or external—in dental abscesses, pyorrhœa alveolaris, necrosis, in bleaching teeth, as a cleanser, a disinfectant or as a hæmostatic.

These preparations have been thoroughly tested, and their value attested to by such reliable men as Drs. Ottolengui, Bodecker, Chas. B. Atkinson, M. L. Rhein and others. What I have to say is based upon their published records, partially confirmed in my own experience.

Pyrozone is manufactured in three grades: (1) medicinal pyrozone, 3 per cent. or 15 volume aqueous solution of hydrogen peroxide; (2) antiseptic pyrozone, a 5 per cent. solution in ether, and (3) caustic pyrozone, a 25 per cent. etherial solution.

(1) Medicinal pyrozone has about the same range as peroxide of hydrogen, but with the advantage of absolute stability and exact strength. It is a rapidly-acting, non-acid, odorless antiseptic and pus destroyer. Injected into abscesses, or sprayed upon ulcerating surfaces, it decomposes pus with great rapidity, causing instant effervescence, bubbling and frothing as long as any pus or extraneous organic matter remains. It is also a good bleacher, though less penetrating than the stronger grades. When it is desired to be absolutely aseptic, Dr. Rhein dissolved 1¾ grains mercuric bichloride in 2 ounces pyrozone, 3 per cent., which can be used to great advantage in cleansing septic root canals. Being non-toxic, medicinal pyrozone may safely be freely used as a mouth wash.

Dr. C. B. Atkinson considers it of special value in the treatment of teeth with pitted or wasted enamel, as it both bleaches the teeth and retards the destructive work of dental decay. In regard to its stability, Dr. Rhein says "it does not seem to lose its stability after any length of time."

(2) Antiseptic pyrozone, a 5 per cent. solution $H_2O_2$ in ether, is a more powerful antiseptic, which acts on pus with great energy, the rapid evaporation of ether leaving the concentrated $H_2O_2$ behind. It is very useful in removing the green and brown stains about the necks of children's teeth, and in bleaching teeth, applying it on a pledget of cotton. If the soft tissues are accidentally touched, apparent coagulation occurs, but the tissues soon resume their natural color, with no eschar or slough and no permanent change of tissue. This 5 per cent. solution is of great value in sterilizing about bridges and crowns, the rapid evaporation of the ether aiding in procuring the perfect dryness so essential in using the soft cements. It may also be used to spray cavities of decay when ready for filling, sterilizing and drying the cavity at the same time. It is also valuable as a spray on ulcerating surfaces, apthous patches, congested fauces, etc. Great care must be taken not to allow it to touch an exposed pulp, as it expands the tissue and would cause severe pain.

It must be borne in mind that pyrozone is death to all extraneous organic matter, whether as pus or in a flow of venous blood, contact with either of which will produce some degree of effervessence and froth. It is an absolute indicator of pus; then *only* after the territory has been bathed and rinsed and made clean. Subsequent effervessence may then be considered an indication of pus.

(3) The 25 per cent. pyrozone is pronounced by Dr. Charles B. Atkinson "probably the best bleacher of teeth that has ever been offered." He says: "Its effect is exceedingly prompt, and the results are permanent." In deep pockets of pyorrhœa alveolaris a small tent wet with the 25 per cent. will, in most cases, terminate the suppuration. Is also valuable in root canals with putrescent pulps. In using the caustic grade care should be taken that no excess oozes from the tampon. Any excess should be removed by bibulous paper. Tannin and glycerine will relieve the pain and burning caused by accidental action upon healthy soft tissues. This burning is more severe on healthy than upon diseased tissue.

Of Weld's syrup of iron chloride (1 fluid ounce=24 m. tri. ch. iron, officinal), I will only say that in this palatable preparation the disastrous effects of the tincture of chloride of iron upon the teeth have been practically overcome, the free acid being neutralized, while the therapeutic value is unimpaired. It is easily assimilated, does not derange the stomach nor produc nausea or disagreeable eructations. It will not injure the enamel of the teeth, though a slight stain, similar to that made by soft-boiled eggs, may be produced. It is not really a *syrup* in the usual sense.

Another easily administered form of iron is found in the bipalatinoids of the New York Quinine and Chemical works.

In this form, the agents to be combined are contained in the two halves of a double convex capsule, but kept apart by a soluble air-tight partition until the capsule is dissolved in the stomach, when chemical reaction takes place.

In the *ferrous carbonate* bi-palatinoid, for instance, one grain of pure powdered dessicated sulphate of iron and two-third grains carbonate of soda are enclosed each in an air-tight compartment of the double capsule. When this is dissolved in the stomach molecular reaction takes place, and the *green ferrous carbonate* results, the chemical form of iron most easily absorbed by the system. Dr. Chas. B. Atkinson considers this method of administering iron tonics far superior to any liquid preparation. Various other combinations of iron are furnished.—*Southern Dental ournal.*

